# Processing speed and the relationship between Trail Making Test-B performance, cortical thinning and white matter microstructure in older adults

**DOI:** 10.1016/j.cortex.2017.07.021

**Published:** 2017-10

**Authors:** Sarah E. MacPherson, Simon R. Cox, David A. Dickie, Sherif Karama, John M. Starr, Alan C. Evans, Mark E. Bastin, Joanna M. Wardlaw, Ian J. Deary

**Affiliations:** a Centre for Cognitive Ageing and Cognitive Epidemiology, University of Edinburgh, UK; b Department of Psychology, University of Edinburgh, UK; c Scottish Imaging Network, a Platform for Scientific Excellence (SINAPSE) Collaboration, Edinburgh, UK; d Department of Neuroimaging Sciences, Centre for Clinical Brain Sciences, University of Edinburgh, UK; e Department of Neurology and Neurosurgery, McConnell Brain Imaging Center, Montreal Neurological Institute, McGill University, Montreal, QC, Canada; f Department of Psychiatry, Douglas Mental Health University Institute, McGill University, Verdun, QC, Canada; g Alzheimer Scotland Dementia Research Centre, The University of Edinburgh, Edinburgh, UK

**Keywords:** Aging, Executive function, Neuroimaging, Processing speed, Trail Making Test

## Abstract

Part B of the Trail Making Test (TMT-B) is widely used as a quick and easy to administer measure of executive dysfunction. The current study investigated the relationships between TMT-B performance, brain volumes, cortical thickness and white matter water diffusion characteristics in a large sample of older participants, before and after controlling for processing speed. Four hundred and eleven healthy, community-dwelling older adults who were all born in 1936 were assessed on TMT-B, 5 tests of processing speed, and provided contemporaneous structural and diffusion MRI data. Significant relationships were found between slower TMT-B completion times and thinner cortex in the frontal, temporal and inferior parietal regions as well as the Sylvian fissure/insula. Slower TMT-B completion time was also significantly associated with poorer white matter microstructure of the left anterior thalamic radiation, and the right uncinate fasciculus. The majority of these associations were markedly attenuated when additionally controlling for processing speed. These data suggest that individual differences in processing speed contribute to the associations between TMT-B completion time and the grey and white matter structure of older adults.

## 1 Introduction

The Trail Making Test (TMT) is one of the most commonly used tests of executive function in clinical neuropsychological assessment (Delis et al., 2001, Lezak, 1995, Reitan and Wolfson, 1993). Part A is administered as a baseline measure of motor and visual search speed, whereas Part B is administered as a measure of set-shifting and inhibition (Arbuthnott and Frank, 2000, Gläscher et al., 2012, Kortte et al., 2002, Strauss et al., 2006). Poor performance on TMT-B is often associated with lesions in the dorsolateral prefrontal cortex (Davidson et al., 2008, Moll et al., 2002, Stuss et al., 2001, Yochim et al., 2007, Zakzanis et al., 2005) and the anterior cingulate (Gläscher et al., 2012), although significant differences between frontal and non-frontal patients' TMT-B performance are not always found (Reitan and Wolfson, 1995, Tamez et al., 2011, Chan et al., 2015; see Demakis, 2004, for a meta-analysis).

TMT performance is found to decline, on average, in healthy aging (Giovagnoli et al., 1996, Hamdan and Hamdan, 2009, Hashimoto et al., 2006, Hester et al., 2005, Periáñez et al., 2007, Seo et al., 2006) with performance on TMT-B declining significantly more than TMT-A in older adults (Drane et al., 2002, Rasmusson et al., 1998). Age-related decline in TMT performance has been associated with poorer grey matter volume in the dorsolateral and ventrolateral prefrontal cortex, medial prefrontal cortex, frontal pole, right inferior frontal gyrus, temporal lobe, insular cortex, caudate, globus pallidus, posterior parietal lobe, occipital cortex and the cerebellum (Newman et al., 2007, Pa et al., 2010, Ruscheweyh et al., 2013). In terms of white matter microstructural integrity and TMT performance, while Perry et al. (2009) reported a significant association between age and TMT-B performance strongly mediated by the effect of age on white matter microstructure, Koch et al. (2013) found better white matter microstructure of the body of the corpus callosum was associated with less time needed to complete TMT-A but not TMT-B.

Research has shown that processing speed is significantly associated with performance on the TMT-B in older adults (Oosterman et al., 2010, Salthouse, 2011a). However, processing speed can be assessed in at least three different ways (see Deary, 2000). In terms of psychometric behavioural tests, participants must make simple decisions that would be completed correctly if sufficient time was provided (e.g., Digit Symbol-Coding subtest from the Wechsler Adult Intelligence Scales; Wechsler, 1997). In terms of cognitive-experimental psychology and psychophysics tests, the responses are even less demanding and made more quickly than psychometric tests, with cognitive-experimental tests using simple and choice reaction times to assess processing speed, and psychophysical measures using inspection time. In this study, TMT-A was not administered; instead we derived a latent measure of processing speed as the common variance across five speed of processing tasks including psychometric behavioural, cognitive experimental and psychophysical measures (Deary et al., 2007). In addition, given that some of these processing speed measures might arguably involve more complex decision-making, more akin to executive abilities, we also derived a simple processing speed factor. We examine the extent to which these two simple and complex processing speed measures influence the relationship of TMT-B completion time with cortical thickness and white matter microstructure.

## 2 Methods

### 2.1 Participants

Participants were from the Lothian Birth Cohort 1936 (LBC1936), a longitudinal study of aging of individuals all born in 1936 and living mainly in the Edinburgh and Lothians region of Scotland, UK (Deary et al., 2012, Deary et al., 2007). Participants first had cognitive and other data measured at Wave 1 at age ∼70 years between 2004 and 2007. No participant had a diagnosis of dementia at entry. Wave 2 testing was carried out 3 years later. Wave 1 data are not reported as there was no neuroimaging at Wave 1, and TMT was not administered during Waves 1 or 2. At Wave 3, participants attended cognitive testing and underwent brain MRI at ∼76 years. For the present study, 411 participants (219 males, 192 females) were included who had performed the TMT-B, had brain MRI data, achieved a score of 24 or greater on the Mini-Mental State Examination (MMSE; Folstein, Folstein, & McHugh, 1975), and had a score of less than 11 on the depression subscale of the Hospital Anxiety and Depression Scale (Snaith, 2003) at Wave 3. Cognitive testing and MRI scanning were undertaken on separate occasions with four participants attending their brain MRI scan first (lag = 39.40 days, SD = 30.74, range = −36 to 320). No participant self-reported a diagnosis of dementia either at entry to LBC1936 or Wave 3 (i.e., their current assessment). No one had a history of traumatic brain injury, stroke, brain tumour or other neurological conditions. All participants were healthy older adults who were considered able to live independently. Study approval was granted by the Lothian Research Ethics Committee (LREC/2003/2/39) and the Multi-Centre Research Ethics Committee for Scotland (MREC/01/0/56). Written informed consent was obtained from all participants, and has been kept on file.

### 2.2 Cognitive testing

Details of the full Lothian Birth Cohort 1936 protocol that participants completed are reported in Deary et al. (2007). Here, we describe a subgroup of tasks that were administered at Wave 3 of the protocol and are examined in the current study. Part B of the Trail Making Test (TMT-B) was administered to cohort members using pen and paper, and standard administration instructions (Bowie & Harvey, 2006). The only difference was that all TMT-B completion times were included, rather than a maximum of 300 sec. The domain of processing speed was tested using Symbol Search (WAIS-III; Wechsler, 1997); Digit-Symbol (WAIS-III; Wechsler, 1997); Simple and 4-Choice Reaction Time (Cox, Huppert, & Whichelow, 1993), and visual Inspection Time (Deary et al., 2004), which have been reported previously (Deary et al., 2010, Deary et al., 2007). Briefly, in Symbol Search, participants indicated whether one of two target symbols on the left of a row was also included among the five symbols printed on the right. The total score is the number of correct responses in 120 sec. In Digit-Symbol, participants wrote down symbols below rows of numbers 1–9, according to a number-symbol code. The score is the number of correct symbols written in 120 sec. In the Reaction Time tasks, participants had a button box with 5 buttons numbered from left to right: 1, 2, 0, 3 and 4. For the Simple Reaction Time task, they pressed zero when a zero appeared on an LCD screen and for the 4-Choice Reaction Time task, they pressed the corresponding button when a 1, 2, 3 or 4 was presented on the screen. The scores for both tasks were the mean correct response reaction times. In the visual Inspection Time task, individuals indicated using a button press which of two parallel, vertical lines was longer. In this task, there was no measure of response speed; the correctness of the response was recorded, and there were 15 different stimulus durations, ranging from 6 to 200 msec. Participants were repeatedly instructed to respond accurately rather than quickly and the next stimulus was not presented until a response was made. The score is the total number of correct responses out of 150.

### 2.3 Brain MRI acquisition and processing

Full details of the brain MRI acquisition protocol have been described previously in an open-access article (Wardlaw et al., 2011). Briefly, MRI acquisition was conducted on the same 1.5 T GE Signa Horizon HDx clinical scanner (General Electric, Milwaukee, WI, USA). Acquisition comprised T2-, T2*- and FLAIR-weighted axial scans, and a high-resolution 3D T1-weighted volume sequence acquired in the coronal plane (voxel dimensions 1 × 1 × 1.3 mm). The diffusion MRI protocol consisted of seven T2-weighted, and set of diffusion-weighted (*b* = 1000 sec/mm^2^) axial single-shot spin-echo echo planar volumes acquired with diffusion gradients applied in 64 non-collinear directions (voxel dimensions 2 × 2 × 2 mm).

Intracranial volume, whole brain, grey matter, normal-appearing white matter and white matter hyperintensity (WMH) volumes were measured using a previously described method which exploits multispectral image intensities from T1-, T2-, T2*- and FLAIR-weighted sequences (Valdés Hernández et al., 2010, Wardlaw et al., 2011). We explicitly defined WMH as punctate, focal or diffuse lesions in all subcortical regions, according to Standards for Reporting Vascular changes on nEuroimaging (STRIVE; Wardlaw et al., 2013). Periventricular and deep WMH were not considered separately, as some previous research has demonstrated they are highly correlated (e.g., DeCarli, Fletcher, Ramey, Harvey, & Jagust, 2005, reported a correlation around *r* = .9) and often spatially proximal. Infarcts (including lacunar infarcts) and enlarged perivascular spaces were excluded from the WMH masks. WMH are a common finding in imaging of healthy older adults (see Muñoz Maniega et al., 2015, Wardlaw et al., 2015) and the majority of participants in this study exhibited at least some WMH volume (395 out of 411). All segmentations were checked for accuracy and manually edited, blind to all other participant information.

Cortical thickness was measured using CIVET 1.1.12 based on participants' Wave 3 MRI scans. CIVET is a fully-automated pipeline developed at the Montreal Neurological Institute (http://www.bic.mni.mcgill.ca; Ad-Dab'bagh et al., 2006, Zijdenbos et al., 2002) using the following processing steps (Karama et al., 2009, Karama et al., 2015): (1) registration of T1-weighted volumes to an age-specific template; (2) bias field (intensity non-uniformity) correction; (3) brain extraction; (4) segmentation of grey and white matter, and cerebrospinal fluid; (5) definition of cortical thickness at 81,924 vertices (the perpendicular distance between grey and white matter surfaces) across the cortex via the t-link metric; (6) inverse of registration at step 1 for cortical thickness measurements in the native space of each subject; and (7) smoothing with a 20-mm kernel. Visual inspection of the CIVET outputs was conducted blind to subject characteristics. Four hundred and three subjects had 3D T1-weighted volume scans for cortical thickness processing. Of these, 47 subjects (11.7%) failed processing due to motion artefact/poor scan quality, leaving 356 subjects for cortical thickness statistical analysis. This is within expected rates of failure in cortical thickness processing (Ducharme et al., 2016).

Diffusion MRI data were also available for 389 out of 411 participants at Wave 3. To quantify white matter tract microstructure, diffusion MRI data was initially pre-processed to extract brain, remove bulk patient motion and eddy current-induced artefacts. Parametric maps of fractional anisotropy (FA) and mean diffusivity (MD) were generated for every participant using freely-available tools in FSL (FMRIB, Oxford, UK: http://www.fmrib.ox.ac.uk). While FA and MD are derived from the same raw water molecular diffusion data, they are widely perceived to provide different information about the underlying microstructure of white matter. The directional coherence of water diffusion is considered to denote the degree to which factors including (but not restricted to) myelination impede cross-fibre diffusion, whereas MD provides a general index of the overall magnitude of water diffusion (irrespective of its direction) and is more informative of extra-cellular water content which may be caused by differences in blood–brain barrier integrity, for example (Cox et al., 2016). Probabilistic neighbourhood tractography (PNT), as implemented in the TractoR package (http://tractor-mri.org.uk; Clayden et al., 2011), was used to segment tracts of interest automatically with the BedpostX/ProbTrackX algorithm run with a two-fibre model and 5000 streamlines per seed point providing the underlying connectivity data. Tract-averaged FA values (weighted by connection probability) where then determined for the following 12 tracts: genu and splenium of the corpus callosum, bilateral anterior thalamic radiation (ATR), cingulum bundle, arcuate fasciculus, uncinate fasciculus, and inferior longitudinal fasciculus (ILF). Following visual inspection, blind to participant characteristics, those tract masks that exhibited aberrant or truncated pathways or were not anatomically plausible representations of the tract-of-interest were excluded, leaving 345–388 instances of each tract for analysis.

### 2.4 Statistical analysis

All statistical analyses were performed in SPSS version 22.0 (SPSS Inc, Chicago, IL, USA), except for cortical thickness analyses which were run with SurfStat MATLAB toolbox (http://www.math.mcgill.ca.keith/surfstat) for Matrix Laboratory (MATLAB) R2014a (© 1994–2014 The MathWorks, Inc.). A general factor of processing speed was derived by entering all five processing speed measures into a principal component analysis (PCA) and extracting the first unrotated principal component using the Principal components extraction method (see Corley et al., 2010, Luciano et al., 2009). The first unrotated component explained 52.43% of the variance, and all component loadings were >.60 and the scree plot clearly indicated the extraction of a single component. This complex processing speed factor contains variance that is common to all five processing speed tests. Based on the rationale of content validity, we also derived a simple processing speed variable, using the two processing speed tests not thought to involve complex decision-making. As principal component analysis requires more than 2 variables to derive a latent variable, the simple processing speed factor was the standardised mean of *z*-scores of Simple Reaction Time and Inspection Time.

The relationships between brain volumetry measures and TMT-B completion time were examined using linear regressions. Each brain volume measure was entered separately as an independent variable, with sex, education, age in days at scan, and intra-cranial volume as covariates and TMT-B completion time as the dependent variable. We then examined the attenuation of the brain volumetry-TMT-B relationships when simple processing speed was added to the model as a covariate. In a final model, complex processing speed replaced simple processing speed as a covariate. The *p*-values for the TMT-B main effect were corrected for simultaneous comparisons using False Discovery Rate (FDR; Benjamini & Hochberg, 1995).

The associations between mean cortical thickness and cortical thickness at each vertex across the mantle and TMT-B completion time, with age at scan, education, sex and intra-cranial volume as covariates were then modelled. In a second model, we additionally covaried for simple processing speed and then, in a final model, covaried for complex processing speed. The significance of results for cortical thickness were corrected for multiple comparisons using Random Field Theory (RFT) to avoid false positives when more than 80,000 tests were performed (Brett et al., 2004, Worsley et al., 1992). They were then displayed on the average grey matter surface. Cluster *p*-values show regions of connected vertices with *p*-values below .001 in clusters whose extent is significant at *p* < .05 (http://www.math.mcgill.ca.keith/surfstat), i.e., a collection of connected vertices with *p* < .001 that was unlikely to occur by chance. Vertex *p*-values show individual vertices where individual *t* scores are above the vertex-wise RFT critical *t*-value, i.e., statistically significant (*p*_*RFT*_ < .05), which is derived via the expected Euler characteristic (EC ≈ critical *p* value [.05]) and number of resolution elements (“resels”) in the *t* cortical map (Brett et al., 2004, Worsley et al., 1992).

We derived a global FA score by entering the FA values for the 12 tracts into a principal component analysis and calculating the regression scores for the first unrotated principal component. The first unrotated component explained 34.87% of the variance, and all component loadings were >.43. We also derived a global MD score from the first unrotated solution from a principal component analysis of the MD values from the 12 tracts. The first unrotated component explained 43.07% of the variance, and all component loadings were >.30. We examined relationships between TMT-B completion time and the global FA and MD scores, as well as tract-specific FA and MD using linear regressions. Each white matter microstructure measure was entered individually as an independent variable, with sex, education, age in days at scan, intra-cranial volume, and WMH volume as covariates and TMT-B completion time as the dependent variable. Then the attenuation of the relationships between the tract-specific FA and MD measures and TMT-B completion time was examined when including simple and complex processing speed in the models. Simple and complex processing speed were entered as covariates in models 2 and 3 respectively. The *p*-values for the TMT-B main effect were corrected for multiple comparisons using FDR. For additional analyses of the relationships between TMT-B performance and processing speed when controlling for age 11 IQ, see Supplementary Material.

## 3 Results

Summary statistics for the demographic and neuropsychological variables are shown in Table 1. The majority of our older adults did not commit more than one error on TMT-B (336 out of 411). Therefore, only the completion times are presented here. The analyses of the TMT error scores are reported in Supplementary Material. Correlations among the processing speed tests and TMT-B performance are reported in Table 2.

**Table 1:** Participant characteristics, including brain volumetry measures.

Characteristic	*N*	M (SD)	Min, max
Age at cognitive testing	411	76.32 (.65)	74.75, 77.75
Age at MRI scan	411	76.43 (.64)	74.90, 77.79
Full-time education	411	10.83 (1.15)	9, 14
MMSE (out of 30)	411	28.80 (1.32)	24, 30
HADS-D (out of 21)	411	2.69 (2.22)	0, 10
Symbol search (total number correct)	410	24.97 (6.28)	5, 53
Digit-symbol substitution (total number correct)	410	55.12 (12.26)	15, 89
Simple reaction time (correct response RTs in seconds)	411	.28 (.05)	.19, .58
4-choice reaction time (correct response RTs in seconds)	411	.67 (.10)	.46, 1.14
Inspection time (out of 150)	401	110.53 (12.34)	49, 136
Simple processing speed factor	401	0 (1)	−2.00, 4.00
Complex processing speed factor	399	0 (1)	−3.72, 2.90
TMT-B (time to complete in seconds)	411	99.77 (50.28)	39, 482
TMT-B (errors)	411	.73 (1.05)	0, 6
Intracranial volume (cm^3^)	396	1440.56 (139.84)	1065.72, 1854.72
Whole brain volume (cm^3^)	396	977.84 (92.69)	713.42, 1248.63
Grey matter volume (cm^3^)	392	467.12 (44.33)	361.35, 593.70
NAWM volume (cm^3^)	392	466.42 (54.38)	280.34, 652.29
WMH volume (cm^3^)	395	15.12 (14.19)	.35, 91.86
Mean cortical thickness	348	3.13 (.16)	2.62, 3.78

MMSE = Mini Mental-State Examination; HADS-D = Hospital Anxiety and Depression Scale-Depression; RTs = response times; TMT-B = Trail Making Test Part B; NAWM = normal-appearing white matter; WMH = white matter hyperintensity.

Simple processing speed factor = Simple Reaction Time and Inspection Time.

Complex processing speed factor = Symbol Search, Digit-Symbol, Simple and 4-Choice Reaction Time and Inspection Time.

**Table 2:** Cognitive test score correlations with N in parentheses.

	1.	2.	3.	4.	5.	6.
1. TMT-B (time to complete in seconds)						
2. TMT-B (total errors)	.37^∗^ (411)					
3. Symbol search	−.52^∗^ (410)	−.19^∗^ (410)				
4. Digit-symbol	−.59^∗^ (410)	−.24^∗^ (410)	.63^∗^ (409)			
5. Simple reaction time	.36^∗^ (411)	.18^∗^ (411)	−.26^∗^ (410)	−.33^∗^ (410)		
6. 4-choice reaction time	.51^∗^ (411)	.16^∗∗^ (411)	−.47^∗^ (410)	−.52^∗^ (410)	.44^∗^ (411)	
7. Inspection time	−.36^∗^ (401)	−.16^∗∗^ (401)	.34^∗^ (400)	.35^∗^ (400)	−.22^∗^ (401)	−.32^∗^ (401)

TMT-B = Trail Making Test Part B; ^∗^*p* < .001; ^∗∗^*p* < .005.

### 3.1 Brain volumetry

For the descriptive characteristics of the brain volumetry measures see Table 1. For each of our linear regression models, we examined multi-collinearity using the variance inflation factor (VIF). In all instances, VIF was below 2, suggesting that there were not high intercorrelations among our predictor variables. The standardised betas and *p*-values for the linear regression analyses involving the brain volumetry measures are displayed in Table 3. Before entering processing speed into the model, TMT-B completion times were significantly associated with whole brain (standardised beta = −.080; 95% CI [−.112, −.048]), grey matter (−.148; 95% CI [−.198, −.085]), normal-appearing white matter (−.072; 95% CI [−.127, −.026]) and white matter hyperintensity volumes (.132; 95% CI [.031, .226]), but not intracranial volume. After adding simple processing speed, TMT-B completion time was associated with whole brain (−.059; 95% CI [−.096, −.023]) and grey matter (−.109; 95% CI [−.165, −.037]) volumes, with percentage attenuation in the standard beta values for normal-appearing white matter and white matter hyperintensity volumes of 60% and 78% respectively. However, TMT-B completion times were no longer associated with any brain volumes when complex processing speed was added (with percentage attenuation in the standard beta values of 73% and 57% for whole brain and grey matter volumes respectively). These magnitudes were significantly mediated when introducing complex processing speed for whole brain volume, normal appearing white matter volume and white matter hyperintensity volume (*Z* ≥ 2.20, *p* < .05).

**Table 3:** The results obtained from linear regression models examining the relationship between brain volumetry measures and TMT-B completion time with and without simple and complex processing speed.

	TMT-B		+Simple		+Complex	
	*β*	*p*	*β*	*p*	*β*	*p*
Intracranial volume (cm^3^)	−.024	.539	−.014	.747	.031	.543
Whole brain volume (cm^3^)	**−.080**	**.0001**	**−.059**	**.001**	−.022*	.302
Grey matter volume (cm^3^)	**−.139**	**.0001**	**−.099**	**.002**	−.060	.107
NAWM volume (cm^3^)	**−.075**	**.003**	−.030	.283	.039*	.218
WMH volume (cm^3^)	**.132**	**.010**	.029	.611	−.064*	.326

*β* = standardised regression coefficient; NAWM = normal-appearing white matter; WMH = white matter hyperintensity; Simple = Controlling for Simple Reaction Time and Inspection Time; Complex = Controlling for Symbol Search, Digit-Symbol, Simple and 4-Choice Reaction Time and Inspection Time; Bold = significant *p*-values after FDR correction based on the actual *p*-values produced; *standardized beta values significantly attenuated (*p* < .05).

### 3.2 Cortical thickness

TMT-B completion times were significantly associated with mean cortical thickness (−.216, *p* = .0001; 95% CI [−.315, −.108]). Even after adding simple (−.184, *p* = .003; 95% CI [−.295, −.063]) or complex (−.181, *p* = .010; 95% CI [−.309, −.042]) processing speed, TMT-B completion time continued to be associated with mean cortical thickness.

**Fig. 1:**
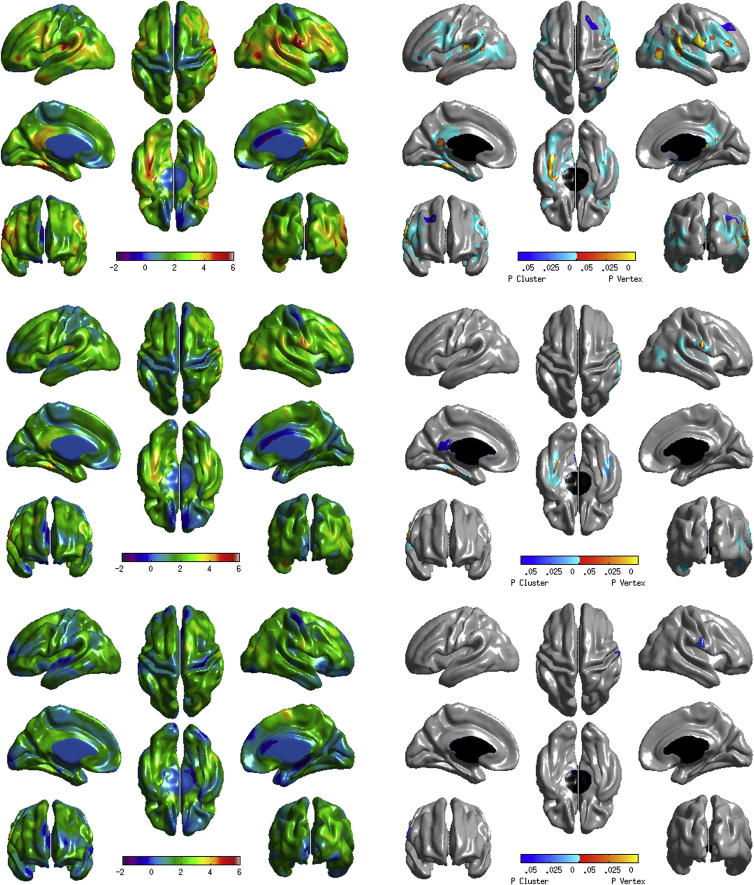
Associations (*t*-maps [left] and *p*-maps [right]) between cortical thickness and Trail Making B completion time corrected for age, education, sex and intracranial volume (Top), age, education, sex, intracranial volume and a latent processing speed factor including inspection time and simple reaction time only (Middle), and additionally corrected for a latent factor of processing speed including all five processing speed measures (Bottom). Cluster *p*-values show regions of connected vertices with *p*-values below .001 in clusters whose extent is significant at *p* < .05 (http://www.math.mcgill.ca.keith/surfstat), i.e., a collection of connected vertices with *p* < .001 that was unlikely to occur by chance. Vertex *p*-values show individual vertices where individual *t* scores are above the vertex-wise RFT critical *t*-value, i.e., statistically significant (*p*_*RFT*_ < .05), which is derived via the expected Euler characteristic (EC ≈ critical *p*-value [.05]) and number of resolution elements (“resels”) in the *t* cortical map (Brett et al., 2004, Worsley et al., 1992).

When controlling for age, gender, education and intra-cranial volume, significant (RFT-corrected) associations were found between TMT-B completion time and cortical thickness in five large and statistically significant (*p*_RFT_ cluster < .05) clusters with a total span of 18,122 vertices; and 2728 individually significant vertices (*p*_RFT_ vertex < .05). These spanned lateral frontal and temporal regions, the Sylvian fissure/insula, supramarginal and inferior parietal regions, inferior motor and sensory areas and the isthmus of the cingulate gyrus (all bilateral). The thinner the cortex in these regions, the slower the older adults were to complete the TMT-B (see Fig. 1, top). When additionally controlling for simple and then complex processing speed, the majority of these associations were attenuated to at least some degree. When simple processing speed (consisting of only Simple Response Time and Inspection Time) was included in the model, small and statistically significant clusters remained across right lateral frontal and temporal lobe, right inferior parietal cortex, and right inferior motor and sensory cortex, left isthmus of the cingulate, and bilateral inferior temporal lobe with a total span of 2483 vertices (86.30% attenuation) and 173 individually statistically significant vertices (93.66% attenuation); associations across the left lateral surface were notably absent (Fig. 1, middle). When complex processing speed (including all five processing speed measures) was included in the model, there was one very small cluster with a span of 129 vertices in the right inferior sensory cortex (99.29% attenuation), and no individually significant vertices (100% attenuation; Fig. 1, bottom); see Supplementary Material for the proportion of the association between cortical thickness and TMT-B completion time accounted for by complex processing speed. This suggests that a general measure of processing speed accounts for most of the associations between TMT-B completion time and cortical thickness, but that selective, mainly right-sided, regions still exhibit TMT-B completion time–thickness associations when accounting for only simple processing speed.

### 3.3 White matter microstructure

Table 4 shows the means and standard deviations for tract-averaged FA and MD for the 12 white matter tracts. Again, the VIF for each of our linear regression models was below 2.

**Table 4:** The total number of tracts available for analysis post-inspection (maximum = 389) and the mean, standard deviation (SD), minimum and maximum values for tract-averaged fractional anisotropy (FA) and mean diffusivity (MD) for the 12 fasciculi-of-interest.

Tract	*N*	FA		MD	
		M (SD)	Min, max	M (SD)	Min, max
Genu	369	.38 (.04)	.25, .51	849.96 (81.99)	631.47, 1126.08
Splenium	373	.51 (.07)	.25, .63	848.56 (153.84)	662.44, 1552.68
Left arcuate	381	.44 (.04)	.27, .54	695.86 (58.08)	583.51, 1043.69
Right arcuate	347	.41 (.04)	.29, .51	677.80 (54.73)	565.29, 892.84
Left ATR	360	.33 (.03)	.24, .44	791.46 (64.15)	603.75, 964.66
Right ATR	381	.34 (.03)	.21, .43	787.65 (81.82)	599.81, 1097.35
Left cingulum	379	.44 (.05)	.25, .57	671.48 (45.14)	558.22, 823.87
Right cingulum	378	.41 (.05)	.27, .52	662.00 (40.44)	534.22, 778.08
Left uncinate	345	.34 (.03)	.24, .43	791.01 (54.55)	654.54, 979.26
Right uncinate	374	.33 (.03)	.19, .41	794.78 (57.53)	629.06, 1320.70
Left ILF	385	.39 (.05)	.20, .51	822.03 (142.85)	673.87, 1698.22
Right ILF	388	.38 (.05)	.18, .51	799.89 (128.18)	619.83, 1790.08
Global^a^	261	0 (1)	−2.29, 2.63	0 (1)	−2.84, 2.58

ATR = anterior thalamic radiation; ILF = inferior longitudinal fasciculus.

a Standardized score from the first unrotated solution from a principal component analysis of FA values from 12 tracts and the MD values from 12 tracts.

The standardised betas and *p*-values for the linear regression analyses involving the FA and MD white matter integrity measures are presented in Table 5. In terms of FA values, TMT-B completion times were significantly associated with the integrity of the right uncinate (−.177; 95% CI [-.272, −.070]). When simple processing speed was added to the model, TMT-B completion time remained associated with the integrity of the right uncinate (−.184; 95% CI [−.294, −.066]) but not when complex processing speed was added to the model (with a percentage change in the standardised beta value of 11%). However, no magnitudes were significantly mediated by processing speed (*p* > .05).

**Table 5:** The results obtained from linear regression models examining the relationship between TMT-B completion time and tract-averaged fractional anisotropy (FA) and mean diffusivity (MD) in the twelve fasciculi-of-interest before and after inclusion of simple and complex processing speed.

Tract	FA						MD					
	TMT-B		+Simple		+Complex		TMT-B		+Simple		+Complex	
	*β*	*p*	*β*	*p*	*β*	*p*	*β*	*p*	*β*	*p*	*β*	*p*
Genu	−.120	.027	−.134	.025	−.100	.152	.097	.061	.118	.039	.074	.268
Splenium	−.114	.036	−.126	.038	−.087	.222	.048	.381	.098	.105	.097	.178
Left arcuate	−.059	.229	−.033	.547	.014	.831	.043	.298	.024	.606	−.013	.814
Right arcuate	.046	.382	.063	.281	.078	.257	.016	.716	−.010	.842	−.028	.626
Left ATR	−.110	.038	−.083	.151	−.079	.249	**.160**	**.001**	.133	.014	.091	.148
Right ATR	−.057	.274	−.022	.705	−.008	.901	.040	.439	.003	.955	−.035	.598
Left cingulum	−.097	.076	−.075	.214	.006	.928	.119	.026	.138	.022	.081	.247
Right cingulum	−.112	.040	−.120	.046	−.081	.253	.093	.073	.102	.078	.037	.586
Left uncinate	−.145	.010	−.117	.060	−.107	.145	.080	.138	.055	.353	.016	.812
Right uncinate	**−.177**	**.001**	**−.184**	**.002**	−.158	.024	.125	.016	.122	.036	.061	.368
Left ILF	−.038	.471	.028	.635	.041	.551	.026	.626	−.009	.882	−.027	.698
Right ILF	−.059	.261	−.004	.940	.039	.572	−.053	.315	−.059	.318	.080	.246
Global	−.146	.014	−.133	.044	−.116	.132	.116	.031	.101	.087	.075	.282

*β* = standardised regression coefficient; ATR = anterior thalamic radiation; ILF = inferior longitudinal fasciculus; Simple = Controlling for Simple Reaction Time and Inspection Time; Complex = Controlling for Symbol Search, Digit-Symbol, Simple and 4-Choice Reaction Time and Inspection Time; Bold = significant *p*-values after FDR correction based on the actual *p*-values produced; *standardized beta values significantly attenuated (*p* < .05).

In terms of MD values, TMT-B was significantly associated with the integrity of the left ATR (.160; 95% CI [.062, .247]). However, when adding simple processing speed and complex processing speed to the model, TMT-B was no longer associated with the white matter microstructure of the left ATR (percentage attenuation of standardised *β* of 17% and 43% respectively). However, these magnitudes for the left arcuate were not significantly mediated by processing speed.

## 4 Discussion

In this study, we have examined the relationships between TMT-B completion time and brain volumetry measures, cortical thickness and white matter microstructure in a group of 411 similar-aged healthy older adults. Differences in TMT-B completion time were significantly associated with a range of volumetric, water diffusion and cortical thickness parameters in this large older sample. Importantly, we demonstrated that these associations between a test traditionally thought to tap executive function and various brain MRI biomarkers were largely reduced when processing speed was entered into the model, which supports prior suggestions that TMT-B is highly dependent upon speed in our healthy, community dwelling sample of older adults (Oosterman et al., 2010, Salthouse, 2011a, Salthouse, 2011b, Salthouse et al., 2000, Sánchez-Cubillo et al., 2009).

Slower TMT-B completion times were associated with smaller whole brain, grey matter and normal-appearing white matter volumes as well as larger white matter hyperintensity volumes; however, these relationships were no longer significant when complex processing speed was entered into the models. In terms of cortical thickness, slower TMT-B completion times were associated with thinner cortex in the frontal and temporal regions, the Sylvian fissure/insula,^1^ and the inferior parietal lobe. When simple processing speed was entered into the model, smaller, significant clusters in similar regions were found but these were no longer significant when complex processing speed was entered into the model (only a very small cluster in the right post-central gyrus was significant). Finally, in terms of white matter microstructure, ostensibly ‘healthier’ integrity in the right uncinate (FA) and left ATR (MD) were associated with faster TMT-B completion times; however, entering simple (in the case of the left ATR) or complex (in the case of the right uncinate and left ATR) processing speed into the models resulted in the removal of these relationships.

When we examined whether these relationships are *significantly* attenuated by the inclusion of processing speed, only the relationships between TMT-B completion time and certain brain volumetry measures (i.e., whole brain, normal appearing white matter and white matter hyperintensity volumes) were significantly reduced by the inclusion of complex processing speed. The attenuations in the relationships between TMT-B and cortical thickness and the white matter tracts were marked, but not significant, when simple or complex processing speed were entered into the model. However, it should be noted that for a number of measures, the percentage change in the standardised beta values was in the same direction, which suggests that there is a marked effect of processing speed on the relationships between TMT-B completion time and our brain measures. Therefore, speed of processing accounts for a substantial amount of the associations between TMT-B completion time and the brain structural indices examined here, at least when accounting for complex processing speed.

The current paper is contrary to work demonstrating the sensitivity of TMT-B errors to frontal lobe damage (Kopp et al., 2015, Stuss et al., 2001). Our analyses did not reveal any significant associations between cortical thickness or white matter tract integrity and TMT-B error scores (see Supplementary Material). This is not surprising given that TMT-B errors are less common in healthy adults, who typically make only one error, if they make an error at all (Ashendorf et al., 2008, Ruffolo et al., 2000). In the current study, 82% of our participants made ≤ one error. We would argue that our findings remain of clinical and theoretical importance given that the TMT is frequently administered to older adults with and without neurological conditions.

Recent research has demonstrated the importance of the medial orbital frontal cortex and rostral anterior cingulate in TMT-B completion times, but not processing speed (Nestor et al., 2015, Ohtani et al., 2017). However, processing speed was based either on TMT-A (Ohtani et al., 2017) or the combination of the Digit Symbol-Coding and Symbol Search subtests of the WAIS (Nestor et al., 2015). Importantly, our study controlled for different types of processing speed using five processing speed tests involving three levels of description: psychometric behavioural, cognitive experimental and psychophysical measures of processing speed (Deary et al., 2007). The above analyses demonstrated that TMT-B completion time significantly correlated with all five processing speed measures (correlation coefficients ranging between .36 and .59) and all five measures significantly correlated with one another. However, it may be that some of these processing speed tasks involve decision-making processes more similar to executive abilities (e.g., Symbol Search, Digit-Symbol, and 4-Choice Reaction Time), and similar to those specifically required for TMT-B. For this reason, we controlled for simple (i.e., Simple Reaction Time and Inspection Time) as well as complex (i.e., all five measures) processing speed in our analyses. As the correlation coefficients for the three complex processing speed measures did not significant differ from one another (*p* ≥ .10) but they were significantly higher than the correlation coefficients for Simple Reaction Time and Inspection Time (*p* < .008), it could be argued that our “simple” processing speed measure (which did not comprise sufficient manifest variables to employ data reduction) merely provided a weaker index of the component score using all 5 measures. However, even controlling for simple processing speed using these tests with weaker correlations resulted in attenuations in some of our brain–cognition relationships.

While we used an appropriate control for type I error, the significant relationship between TMT-B completion time and a small cluster in the right inferior somatosensory cortex (after controlling for complex processing speed) should be interpreted with caution, particularly given that it contained no individually significant vertices. Another explanation may be that our older adults were articulating their responses while performing TMT-B, as the inferior somatosensory region, together with the inferior sensorimotor cortex, are typically activated during orofacial speech movements (Grabski et al., 2012). However, it is surprising that this association is lateralized to the right rather than the left hemisphere. Activation in the right primary somatosensory cortex has been reported in a functional near-infrared spectroscopy study involving the TMT; however greater activation was associated with slower rather than faster TMT completion times in older adults (Hagen et al., 2014). While it is unlikely that the inferior somatosensory region is associated with the executive aspects of TMT-B, articulation may be a small component of TMT-B completion time that is not measured in processing speed measures; it remains unclear what the role of the somatosensory region is in TMT-B completion time.

We note that our detailed analysis of tract-specific microstructure with respect to TMT-B completion time did not include estimates of the entire superior longitudinal fasciculus (SLF). It has previously been suggested that this large fibre bundle may be involved in TMT-B performance (Muir et al., 2015, Perry et al., 2009). However, our tractography method allowed us to only focus on the arcuate portion of this pathway. The arcuate connects the superior temporal lobe and the dorsolateral prefrontal cortex. While both are cortical areas implicated in TMT-B performance, the current data do not allow us to comment on the relative importance of other constituents of the SLF.

In addition to the limitations discussed above, several other aspects of our study should be noted. TMT-A was not administered as part of the LBC1936 protocol and so TMT ratio or proportion scores, which have been discussed as purer executive measures (e.g., Stuss et al., 2001) could not be considered. Also, while there was no self-report of dementia, we cannot conclude that our sample did not include any participants with mild cognitive impairment who might be in the early stages of dementia. Moreover, while the LBC1936 affords access to a large group of healthy older adults, they are a self-selecting group and probably characterise a somewhat restricted sample (e.g., Johnson, Brett, Calvin, & Deary, 2016) and so caution should be taken when relating these findings to the younger and wider older adult population. However, this is also likely to mean that we have smaller-than-expected effect sizes than those we would have found had we a wider distribution. Finally, it should be noted that the findings cannot be directly related to older adults experiencing pathological aging due to neurodegenerative diseases such as Alzheimer's disease. For example, our measure of WMH volumes included both deep and periventricular WMH (because they are very highly correlated in healthy samples), yet WMH location may be less collinear in cognitively impaired samples (Smith et al., 2011), allowing investigation of their differential contributions to TMT-B performance.

Nevertheless, our study strengths include the large sample size and the age homogeneity (which largely reduces the important confounding effect of age). Large birth cohorts provide the opportunity to examine neural correlates with increased statistical power to avoid type II error (false negative) and accurately detect relationships with less need to statistically control for age (Freedman, Pisani, & Purves, 2007). Another strength is our comprehensive multi-modal analysis that allowed us to examine both grey and white matter structure in relation to TMT-B completion time within the same group. We were also able to address important previous criticisms of the TMT-A as an appropriate covariate for processing speed, using a wider battery of processing speed tests and appropriate data reduction techniques. We tentatively highlight the biologically plausible relationship between poorer TMT-B completion times and thinner cortex in the right frontal and lateral temporal lobe as well as the white matter microstructure of the right uncinate fasciculus, the white matter tract connecting these two brain regions. This finding may warrant future study. On the basis of these current data, we can conclude that the grey and white matter correlates of the TMT-B score appear to be highly related to individual differences in processing speed. Regardless, the TMT-B remains an important clinical tool in the diagnoses of different clinical groups, as well as the assessment of disease severity and progression.

## Footnotes

1 Caution should be taken when considering the cortical thickness/cognition association between the insula and TMT-B performance found prior to controlling for processing speed. Cortical thickness estimates of the insula tend not to be very precise using automated cortical thickness pipelines, in part due to a high level of insular gyrification.
