# Supplementary material for: Processing speed and the relationship between Trail Making Test-B performance, cortical thinning and white matter microstructure in older adults
Source: Cortex. 2017 Oct;95:92–103. doi: 10.1016/j.cortex.2017.07.021 (PMC5637162; doi:10.1016/j.cortex.2017.07.021)

**Introduction**

Importantly, our previous work has shown that TMT-B completion times in older age correlate with childhood IQ at age 11 (Dick et al., 2010), and that some brain-cognition associations are substantially attenuated by childhood IQ (Karama et al., 2014). Therefore, any significant brain associations with TMT-B completion time within older age in the present study might be due to long-lasting IQ differences. Processing speed measured in older age also correlates with childhood IQ (Deary, Johnson, & Starr, 2010), and so any attenuation in our within-older-age brain-cognition associations after controlling for processing speed might be due to the relationship between processing speed and long-lasting IQ differences. Most of the LBC1936 sample took the same IQ-type test at about age 11 years. Therefore, we repeated our analyses with both TMT-B and processing speed controlling for age 11 IQ. Here we also include the analyses of TMT-B errors.

**Methods**

**Moray House Test No. 12**

Around the age of 11 years, participants were administered the Moray House Test No. 12 at school as part of the Scottish Mental Survey 1947 (Deary, Whalley, & Starr, 2009; Scottish Council for Research in Education, 1949). The test includes items related to: following directions (14 items); same-opposites (11); word classification (10); analogies (8); practical items (6); reasoning (5); proverbs (4); arithmetic (4); spatial items (4); mixed sentences (3); cipher decoding (2); and other items (4). Participants had 45 minutes to answer the questions with a maximum score of 76 (for more detail see Deary, Whiteman, Starr, Whalley, & Fox, 2004). Childhood IQ scores have been found to be strongly related to education and occupational attainment, and health outcomes in adulthood and later life (Deary et al., 2009).

**Results**

Childhood IQ was entered as a covariate into the regression model in addition to sex, age in days at scan, education and intracranial volume. In terms of the brain volumetry measures, TMT-B completion times remained significantly associated with whole brain (-0.087; 95% CI [-0.134, -0.057]), grey matter (-0.126; 95% CI [-0.212, -0.071]) and normal-appearing white matter (-0.078; 95% CI [-0.148, -0.024]) volumes but not white matter hyperintensity or intracranial volumes (with percentage attenuation in the standard beta value of 22% for white matter hyperintensity). Once simple processing speed was added, TMT-B completion time remained associated with whole brain (-0.070; 95% CI [-0.120, -0.034]) and grey matter (-0.083; 95% CI [-0.172, -0.016]) volumes (with percentage attenuation of 43% in the standard beta value for normal-appearing white matter). However, TMT-B completion times were no longer associated with any brain volumes when complex processing speed was added (with percentage change in the standard beta values of 58% for whole brain and 64% for grey matter volumes). For normal-appearing white matter and white matter hyperintensity volumes, the magnitude was significantly mediated when complex processing speed was introduced (Z > 1.98, p < .05).

- Insert Table S1 around here -

In terms of cortical thickness, 337 participants had age 11 IQ scores and did not fail our quality control due to motion artefacts or poor scan quality. The associations, therefore, between cortical thickness at each vertex across the mantle and TMT-B score, with age at scan, sex, education and intracranial volume as covariates were modelled in this smaller sample. When controlling for childhood IQ, smaller, significant clusters were found in the isthmus cingulate, inferior and anterior temporal and insula loci with a total span of 6145 vertices and 577 individual significant vertices. When simple processing speed (Simple Response Time and Inspection Time) was also included, only three statistically significant clusters remained in the inferior and anterior temporal lobe, with a total span of 1357 vertices (77.92% attenuation) and 76 individually statistically significant vertices (86.83% attenuation). When complex processing speed (all five measures) was included in the model, there were no significant vertices or clusters (100% attenuation). These findings suggest that the effects are markedly attenuated by the inclusion of childhood IQ.

- Insert Figures S1-S3 around here -

In terms of FA values, TMT-B completion times became significantly associated with the integrity of the left uncinate (-0.198; 95% CI [-0.328, -0.077]) instead of the right uncinate when childhood IQ was also entered into the model. The percentage change in the standardised beta values for the integrity of the right uncinate was 21%. When either simple or complex processing speed were added, TMT-B was not associated with white matter microstructure of the right uncinate (percentage attenuation of standardised β = 14% and 24% respectively). In terms of MD values, when childhood IQ was entered into the model, TMT-B continued to be significantly associated with the integrity of the left ATR (0.152; 95% CI [0.047, 0.277]). However, when adding either simple or complex processing speed to the model, TMT-B was no longer significantly associated with the integrity of any tract (percentage attenuation of standardised β of 18% for simple processing speed and 38% for complex processing speed). These magnitudes were not significantly mediated when introducing simple or complex processing speed.

- Insert Tables S2 and S3 around here -

We also conducted our linear regression analyses on the TMT-B errors (0 errors; n = 231 versus  1 errors; n = 180) rather than TMT-B completion time as the dependent variable. TMT-B error score was not significantly associated with any of the brain volumetry measures.

- Insert Table S4 around here -

TMT-B error scores were also not significantly associated with mean cortical thickness in any of the models (standardised beta = -0.030, p = 0.576; with simple processing speed = -0.001, p = 0.984; and complex processing speed = 0.014, p = 0.799). In addition, TMT-B had only 2 significant clusters with only 151 vertices between them around the midline (0.18-0.20%).

The standardised betas and p-values for the linear regression analyses involving the FA and MD white matter integrity measures are presented in Table S5. In terms of FA values, TMT-B error score was not significantly associated with the integrity of any of the white matter tracts. While TMT-B errors became significantly associated with splenium FA when complex processing speed was added to the model (percentage increase in the standardised beta value = 22%), the magnitude of this TMT-B error score-splenium FA association was not significantly changed.

In terms of MD values, TMT-B error score was not significantly associated with the integrity of any white matter tract in any of the models.

- Insert Table S5 around here -

**Discussion**

An advantage of our study was the ability to examine whether long-lasting and stable differences in cognitive abilities (measured using childhood IQ), rather than processing speed, underlie the relationships between TMT-B performance and older age brain structure. We conducted additional analyses where childhood IQ was added to our models. In terms of the brain volumetry measures, childhood IQ only confounded TMT-B associations with white matter hyperintensity volumes. While childhood IQ did attenuate the associations with a thicker cortex, the water molecule diffusion parameters FA and MD remained associated with a faster TMT-B completion time. Indeed, our previous work has shown that controlling for age 11 IQ does not attenuate the negative relationship between white matter hyperintensity volume and older age cognitive ability (Valdés Hernández et al., 2013). However, the fact that these loci were then attenuated to non-significance by the addition of a latent speed variable indicates that those cortical loci related to non-speed aspects of TMT-B are explained by the stable differences in cognitive ability that were present even in childhood.

**References**

Brett, M., Penny, W.D., & Kiebel, S.J. (2004). Introduction to random field theory. In: Frackowiak, R.S.J., Friston, K.J., Frith, C., Dolan, R., Price, C.J., Zeki, S., Ashburner, J., & Penny, W.D. (eds.), *Human Brain Function*. Amsterdam, Elsevier Academic Press. p 867-879.

Deary, I.J., Johnson, W., & Starr, J.M. (2010). Are processing speed tasks biomarkers of cognitive ageing? *Psychology and Aging, 25,* 219-228.

Deary, I.J., Whiteman, M.C., Starr, J., Whalley, L.J., & Fox, H.C. (2004). The impact of childhood intelligence on later life: Following up the Scottish Mental Surveys of 1932 and 1947. *Journal of Personality and Social Psychology, 86(1),* 130-147.

Deary, I.J., Whalley, L.J., & Starr, J.M. (2009). *A lifetime of intelligence: Follow-up studies of the Scottish Mental Surveys of 1932 and 1947.* Washington, DC: American Psychological Association.

Dick, F.D., Bourne, V.J., Semple, S.E., Fox, H.C., Miller, B.G., Deary, I.J., & Whalley, L.J. (2010). Solvent exposure and cognitive ability at age 67: a follow-up study of the 1947 Scottish Mental Survey. *Occupational and Environmental Medicine, 67(6),* 401-407.

Karama, S., Bastin, M.E., Murray, C., Royle, N.A., Penke, L., Maniega, S.M., Gow, A.J., Corley, J., Valdes Hernandez, M.C., Lewis, J.D., Rousseau, M.E., Lepage, C., Fonov, V., Collins, L., Booth, T., Rioux, P., Sherif, T., Adalat, R., Starr, J.M., Evans, A.C., Wardlaw, J.M., & Deary, I.J. (2014). Childhood cognitive ability accounts for associations between cognitive ability and brain cortical thickness in old age. *Molecular Psychiatry, 19,* 555-559.

Scottish Council for Research in Education. (1949). *The trend of Scottish intelligence: A comparison of the 1947 and 1932 surveys of the intelligence of eleven-year-old pupils.* London, United Kingdom: University of London Press.

Valdés Hernández, M.C., Booth, T., Murray, C., Gow, A.J., Penke, L., Morris, Z., Maniega, S.M., Royle, N.A., Aribisala, B.S., Bastin, M.E., Starr, J.M., Deary, I.J., & Wardlaw, J.M. (2013). Brain white matter damage in aging and cognitive ability in youth and older age. *Neurobiology of Aging, 34(12),* 2740-2747.

Worsley, K.J., Evans, A.C., Marrett, S., & Neelin, P. (1992). A three-dimensional statistical analysis for cbf activation studies in human brain. *Journal of Cerebral Blood Flow and Metabolism, 12,* 900-918.

Table S1. The results obtained from linear regression models examining the relationship between the brain volumetry measures and sex, age in days at scan, education, intracranial volume and TMT-B completion time and sex, age in days at scan, education, intracranial volume, TMT-B completion time and childhood IQ with and without simple and complex processing speed.

|  | **TMT-B** | | **+ Childhood IQ** | | **+ Simple** | | **+Complex** | |
| --- | --- | --- | --- | --- | --- | --- | --- | --- |
| **Tract** | *β* | *p* | *β* | *p* | *β* | *p* | *β* | *p* |
| Intracranial volume (cm3) | -0.024 | 0.539 | 0.064 | 0.140 | 0.075 | 0.116 | 0.116 | 0.035 |
| Whole brain volume (cm3) | **-0.080** | **0.0001** | **-0.087** | **0.0001** | **-0.070** | **0.001** | -0.034 | 0.134 |
| Grey matter volume (cm3) | **-0.139** | **0.0001** | **-0.126** | **0.0001** | **-0.083** | **0.019** | -0.050 | 0.222 |
| NAWM volume (cm3) | **-0.075** | **0.003** | **-0.078** | **0.007** | -0.043 | 0.172 | 0.018* | 0.602 |
| WMH volume (cm3) | **0.132** | **0.010** | 0.103 | 0.068 | 0.003 | 0.962 | -0.074* | 0.297 |

*β *= standardised regression coefficient; NAWM = Normal-appearing white matter; WMH = White matter hyperintensity; Bold numbers indicate signiﬁcant TMT-B main effect after FDR correction based on the actual p-values produced; * standardized beta values significantly attenuated (p < .05)

Table S2. The results obtained from linear regression models examining the relationship between tract-averaged fractional anisotropy (FA) in the twelve fasciculi-of-interest and sex, age in days at scan, education, intracranial volume, WMH volume and TMT-B completion time and sex, age in days at scan, education, intracranial volume, WMH volume and TMT-B completion time and childhood IQ, with and without simple and complex processing speed

|  | **TMT-B** | | **+ Childhood IQ** | | **+ Simple** | | **+Complex** | |
| --- | --- | --- | --- | --- | --- | --- | --- | --- |
| **Tract** | *β* | *p* | *β* | *p* | *β* | *p* | *β* | *p* |
| Genu | -0.120 | 0.027 | -0.113 | 0.060 | -0.141 | 0.031 | -0.103 | 0.172 |
| Splenium | -0.114 | 0.036 | -0.124 | 0.040 | -0.139 | 0.037 | -0.107 | 0.169 |
| Left arcuate | -0.059 | 0.229 | -0.031 | 0.565 | -0.017 | 0.771 | 0.037 | 0.593 |
| Right arcuate | 0.046 | 0.382 | 0.036 | 0.531 | 0.038 | 0.551 | 0.06 | 0.415 |
| Left ATR | -0.110 | 0.038 | -0.113 | 0.056 | -0.096 | 0.134 | -0.107 | 0.149 |
| Right ATR | -0.057 | 0.274 | -0.058 | 0.311 | -0.033 | 0.602 | -0.021 | 0.769 |
| Left cingulum | -0.097 | 0.076 | -0.132 | 0.028 | -0.127 | 0.055 | -0.045 | 0.558 |
| Right cingulum | -0.112 | 0.040 | -0.120 | 0.047 | -0.148 | 0.025 | -0.099 | 0.193 |
| Left uncinate | -0.145 | 0.010 | **-0.198** | **0.002** | -0.190 | 0.006 | -0.184 | 0.020 |
| Right uncinate | **-0.177** | **0.001** | -0.140 | 0.019 | -0.153 | 0.020 | -0.134 | 0.077 |
| Left ILF | -0.038 | 0.471 | -0.035 | 0.543 | 0.017 | 0.789 | 0.025 | 0.734 |
| Right ILF | -0.059 | 0.261 | -0.017 | 0.770 | 0.048 | 0.453 | 0.107 | 0.150 |
| Global | -0.146 | 0.014 | -0.128 | 0.054 | -0.125 | 0.082 | -0.111 | 0.185 |

*β *= standardised regression coefficient; ATR = anterior thalamic radiation; ILF = inferior longitudinal fasciculus; Simple = Controlling for Simple Reaction Time and Inspection Time; Complex = Controlling for Symbol Search, Digit-Symbol, Simple and 4-Choice Reaction Time and Inspection Time

Bold numbers indicate signiﬁcant TMT-B main effect after FDR correction based on the actual p-values produced

Table S3. The results obtained from linear regression models examining the relationship between tract-averaged mean diffusivity (MD) in the twelve fasciculi-of-interest and sex, age in days at scan, education, intracranial volume, WMH volume and TMT-B completion time and sex, age in days at scan, education, intracranial volume, WMH volume and TMT-B completion time and childhood IQ, with and without simple and complex processing speed

|  | **TMT-B** | | **+ Childhood IQ** | | **+ Simple** | | **+Complex** | |
| --- | --- | --- | --- | --- | --- | --- | --- | --- |
| **Tract** | *β* | *p* | *β* | *p* | *β* | *p* | *β* | *p* |
| Genu | 0.097 | 0.061 | 0.081 | 0.158 | 0.111 | 0.076 | 0.061 | 0.397 |
| Splenium | 0.048 | 0.381 | 0.039 | 0.523 | 0.089 | 0.184 | 0.090 | 0.250 |
| Left arcuate | 0.043 | 0.298 | 0.051 | 0.263 | 0.036 | 0.470 | -0.003 | 0.958 |
| Right arcuate | 0.016 | 0.716 | 0.013 | 0.787 | 0.003 | 0.954 | -0.031 | 0.623 |
| Left ATR | **0.160** | **0.001** | **0.152** | **0.006** | 0.132 | 0.027 | 0.099 | 0.148 |
| Right ATR | 0.040 | 0.439 | 0.041 | 0.468 | 0.019 | 0.757 | -0.035 | 0.623 |
| Left cingulum | 0.119 | 0.026 | 0.142 | 0.016 | 0.174 | 0.008 | 0.119 | 0.114 |
| Right cingulum | 0.093 | 0.073 | 0.103 | 0.073 | 0.116 | 0.065 | 0.047 | 0.516 |
| Left uncinate | 0.080 | 0.138 | 0.121 | 0.043 | 0.101 | 0.120 | 0.055 | 0.465 |
| Right uncinate | 0.125 | 0.016 | 0.107 | 0.068 | 0.108 | 0.093 | 0.032 | 0.660 |
| Left ILF | 0.026 | 0.626 | 0.056 | 0.338 | 0.032 | 0.614 | 0.003 | 0.968 |
| Right ILF | -0.053 | 0.315 | -0.071 | 0.224 | -0.075 | 0.244 | -0.096 | 0.197 |
| Global | 0.116 | 0.031 | 0.099 | 0.098 | 0.095 | 0.141 | 0.067 | 0.367 |

*β *= standardised regression coefficient; ATR = anterior thalamic radiation; ILF = inferior longitudinal fasciculus; Simple = Controlling for Simple Reaction Time and Inspection Time; Complex = Controlling for Symbol Search, Digit-Symbol, Simple and 4-Choice Reaction Time and Inspection Time

Bold numbers indicate signiﬁcant TMT-B main effect after FDR correction based on the actual p-values produced

Table S4. The results obtained from linear regression models examining the relationship between brain volumetry measures and TMT-B errors with and without simple and complex processing speed.

|  | **Error Score** | | **+ Simple** | | **+ Complex** | |
| --- | --- | --- | --- | --- | --- | --- |
|  | *β* | *p* | *β* | *p* | *β* | *p* |
| Intracranial volume (cm^3^) | 0.046 | 0.232 | 0.05 | 0.209 | 0.059 | 0.141 |
| Whole brain volume (cm^3^) | -0.014 | 0.386 | 0.003 | 0.866 | 0.012 | 0.445 |
| Grey matter volume (cm^3^) | -0.016 | 0.572 | 0.015 | 0.612 | 0.025 | 0.383 |
| NAWM volume (cm^3^) | -0.032 | 0.209 | -0.008 | 0.767 | 0.005 | 0.837 |
| WMH volume (cm^3^) | 0.027 | 0.589 | -0.029 | 0.569 | -0.041 | 0.418 |

*β *= standardised regression coefficient; NAWM = Normal-appearing white matter; WMH = White matter hyperintensity; Simple = Controlling for Simple Reaction Time and Inspection Time; Complex = Controlling for Symbol Search, Digit-Symbol, Simple and 4-Choice Reaction Time and Inspection Time

Table S5. The results obtained from linear regression models examining the relationship between TMT-B error scores and tract-averaged fractional anisotropy (FA) and mean diffusivity (MD) in the twelve fasciculi-of-interest before and after inclusion of simple and complex processing speed

|  | **FA** | | | | | | **MD** | | | | | |
| --- | --- | --- | --- | --- | --- | --- | --- | --- | --- | --- | --- | --- |
|  | **Error Score** | | **+Simple** | | **+Complex** | | **Error Score** | | **+Simple** | | **+Complex** | |
| **Tract** | *β* | *p* | *β* | *p* | *β* | *p* | *β* | *p* | *β* | *p* | *β* | *p* |
| Genu | 0.011 | 0.830 | 0.015 | 0.778 | 0.035 | 0.523 | -0.030 | 0.558 | -0.029 | 0.577 | -0.050 | 0.336 |
| Splenium | 0.118 | 0.025 | 0.125 | 0.022 | 0.144 | 0.008 | -0.040 | 0.449 | -0.019 | 0.725 | -0.036 | 0.508 |
| Left arcuate | 0.005 | 0.920 | 0.028 | 0.570 | 0.038 | 0.443 | 0.011 | 0.776 | -0.004 | 0.923 | -0.013 | 0.746 |
| Right arcuate | 0.005 | 0.915 | 0.012 | 0.824 | 0.006 | 0.916 | -0.003 | 0.943 | -0.019 | 0.672 | -0.016 | 0.713 |
| Left ATR | -0.048 | 0.348 | -0.019 | 0.718 | -0.018 | 0.744 | 0.090 | 0.061 | 0.062 | 0.209 | 0.048 | 0.334 |
| Right ATR | -0.029 | 0.563 | 0.000 | 0.997 | 0.001 | 0.984 | 0.029 | 0.565 | -0.005 | 0.930 | -0.010 | 0.851 |
| Left cingulum | -0.038 | 0.467 | -0.011 | 0.842 | 0.009 | 0.875 | 0.025 | 0.632 | 0.019 | 0.723 | -0.006 | 0.909 |
| Right cingulum | -0.065 | 0.218 | -0.047 | 0.388 | -0.032 | 0.559 | 0.038 | 0.453 | 0.032 | 0.542 | 0.009 | 0.858 |
| Left uncinate | -0.063 | 0.247 | -0.038 | 0.501 | -0.030 | 0.600 | 0.079 | 0.125 | 0.070 | 0.191 | 0.057 | 0.286 |
| Right uncinate | -0.075 | 0.151 | -0.069 | 0.204 | -0.052 | 0.334 | 0.044 | 0.388 | 0.031 | 0.558 | 0.011 | 0.836 |
| Left ILF | 0.020 | 0.696 | 0.049 | 0.348 | 0.045 | 0.398 | 0.010 | 0.853 | 0.001 | 0.983 | 0.000 | 0.995 |
| Right ILF | 0.002 | 0.961 | 0.025 | 0.634 | 0.034 | 0.521 | -0.023 | 0.656 | -0.017 | 0.751 | -0.017 | 0.745 |
| Global | -0.046 | 0.426 | -0.027 | 0.658 | -0.020 | 0.734 | 0.045 | 0.385 | 0.032 | 0.557 | 0.026 | 0.636 |

Note: *β *= standardised regression coefficient; ATR = anterior thalamic radiation; ILF = inferior longitudinal fasciculus; Simple = Controlling for Simple Reaction Time and Inspection Time; Complex = Controlling for Symbol Search, Digit-Symbol, Simple and 4-Choice Reaction Time and Inspection Time; Bold = significant p-values after FDR correction based on the actual p-values produced

Captions for Figures

Figure S1. Proportion of the association between cortical thickness and Trail Making B completion time that is accounted for by complex processing speed. The loci and magnitude of attenuations across the cortex were unchanged when the association was considered without age, sex, education and intracranial volume.

Figure S2. Associations (*t* maps [left] and *P*-maps [right]) between cortical thickness and Trail Making B completion time corrected for age, sex, education, intracranial volume and childhood IQ (Top), age, sex, education, intracranial volume, childhood IQ and a latent processing speed factor including inspection time and simple reaction time only (Middle), and additionally corrected for a latent factor of processing speed including all five processing speed measures (Bottom). Cluster *P*-values show regions of connected vertices with *P*-values below 0.001 in clusters whose extent is significant at *P*<0.05 (<http://www.math.mcgill.ca.keith/surfstat>), i.e., a collection of connected vertices with *P*<0.001 that was unlikely to occur by chance. Vertex *P*-values show individual vertices where individual *t* scores are above the vertex-wise RFT critical *t* value, i.e., statistically significant (*P_RFT_*<0.05), which is derived via the expected Euler characteristic (EC ≈ critical *P*value [0.05]) and number of resolution elements (“resels”) in the *t* cortical map (Worsley et al., 1992; Brett et al., 2004).

Figure S3. Associations (*t* maps [left] and *P*-maps [right]) between cortical thickness and Trail Making B errors corrected for age, sex, education and intracranial volume, and a latent processing speed factor including inspection time and simple reaction time only (Top), and age, sex, education, intracranial volume, and a latent factor of processing speed including all five processing speed measures (Bottom). Cluster *P*-values show regions of connected vertices with *P*-values below 0.001 in clusters whose extent is significant at *P*<0.05 (<http://www.math.mcgill.ca.keith/surfstat>), i.e., a collection of connected vertices with *P*<0.001 that was unlikely to occur by chance. Vertex *P*-values show individual vertices where individual *t* scores are above the vertex-wise RFT critical *t* value, i.e., statistically significant (*P_RFT_*<0.05), which is derived via the expected Euler characteristic (EC ≈ critical *P*value [0.05]) and number of resolution elements (“resels”) in the *t* cortical map (Worsley et al., 1992; Brett et al., 2004).


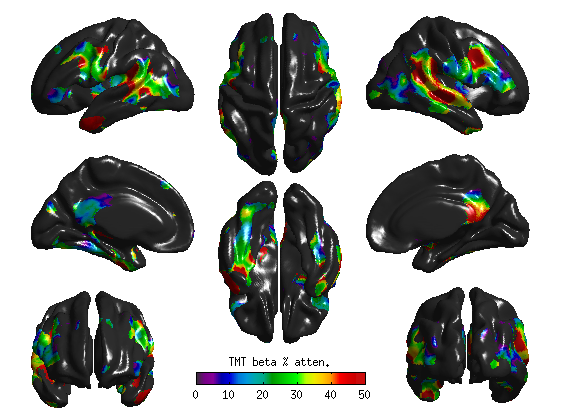


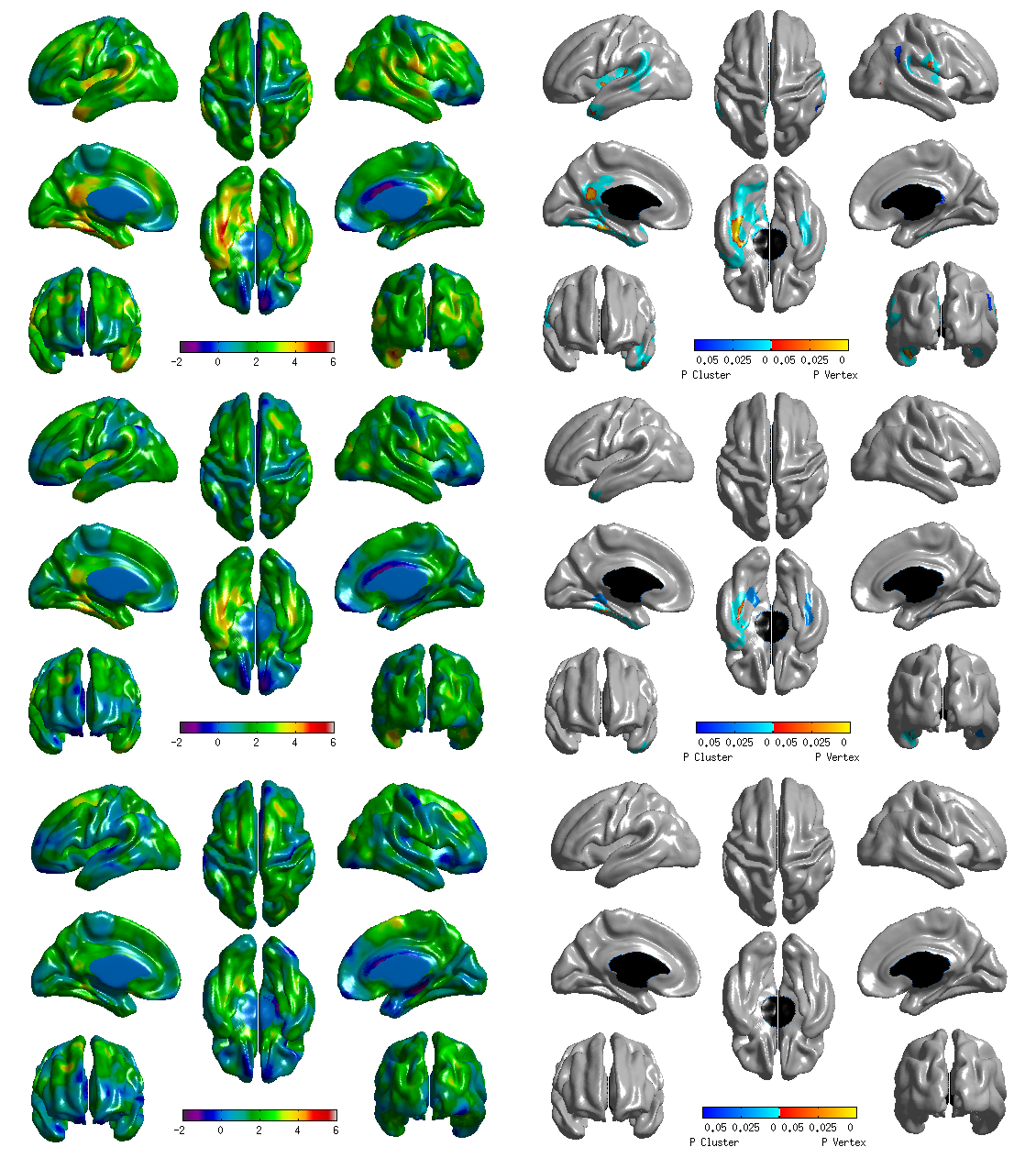


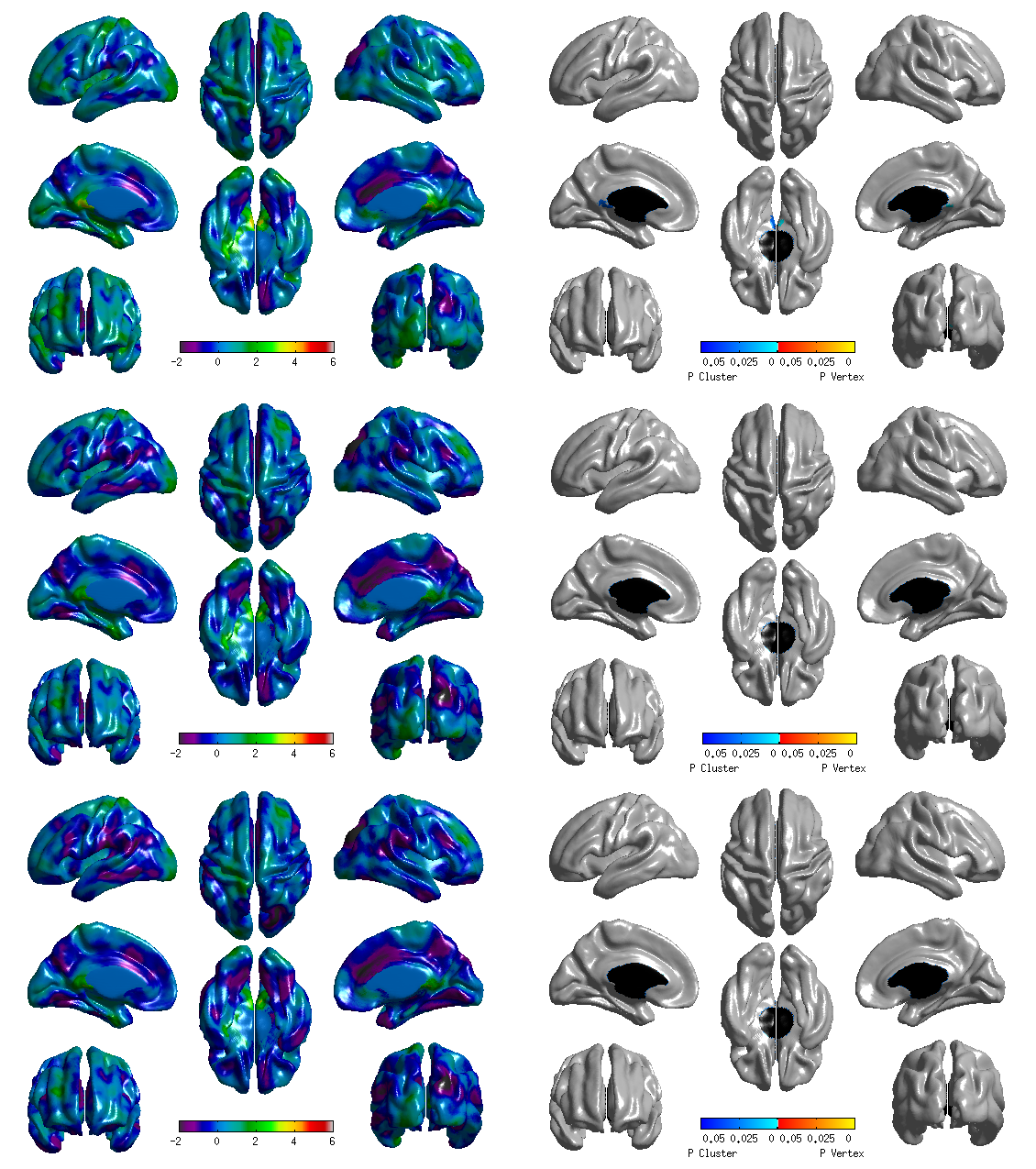

Supplement: Supplementary file 1 [file mmc1.docx]
